# How Valid Is Bandura’s Social Cognitive Theory to Explain Physical Activity Behavior?

**DOI:** 10.3390/ejihpe15020020

**Published:** 2025-02-06

**Authors:** Viktoria Sophie Egele, Eric Klopp, Robin Stark

**Affiliations:** Department of Education, Saarland University, 66123 Saarbrücken, Germany; e.klopp@mx.uni-saarland.de (E.K.); r.stark@mx.uni-saarland.de (R.S.)

**Keywords:** health behavior, validity, self-efficacy, outcome expectations, structural equation model

## Abstract

(1) Background: Although social cognitive theory (SCT) has been widely tested and applied in numerous interventions aimed at optimizing physical activity behavior, the complete theory has rarely been tested in its entirety. Only selected elements have been tested, and specific samples, some of them pathological, have been studied rather than the general population, for whom a lack of physical activity is a huge problem. The present study addresses these two research gaps and tests the tenability of the theoretical assumptions of SCT to explain physical activity behavior in the general population. (2) Methods: A total of 194 German adults (109 male, 85 female) with a mean age of 26.03 years (SD = 10.33) completed two validated questionnaires concerning their expressions on SCT components (t1) and their physical activity (t2). SCT was modeled using a structural equation model with latent variables. (3) Results: The results showed the very good fit of the structural model, indicating that the theoretically stated relations between the constructs in SCT seem to be corroborated, despite some paths seeming to be more important than others. (4) Conclusions: The use of SCT to explain and predict behavior can be seen as justified, even though it once again appears that some aspects (i.e., self-efficacy) are more crucial than others.

## 1. Introduction

Insufficient physical activity is a main cause of non-communicable diseases (e.g., coronary heart diseases and cancer), poor psychological well-being, and premature death (Miko et al., 2020; World Health Organization, 2018). The WHO recommends 150 min of moderate physical activity or 75 min of vigorous physical activity per week, alongside two sessions of additional muscle-strengthening exercise, for adults to achieve considerable health benefits (World Health Organization, 2018). Yet, in a recent long-term study including data from nearly two million people in 168 countries, it was evident that 31.3 percent of adults worldwide do not reach the recommended level of physical activity, and consequently are at increased health risk (Strain et al., 2024). Therefore, it is crucial to understand why individuals engage in physical activity.

Numerous studies have provided evidence that social cognitive theory (SCT) (Bandura, 1986) is suitable for explaining or predicting physical activity behavior (Auster-Gussman et al., 2022; Baird et al., 2021; Heiss & Petosa, 2016; Sebastian et al., 2021; Silveira & Motl, 2019; Stacey et al., 2015; Taylor et al., 2016; Zechner & Gill, 2016). SCT proposes that individuals’ behaviors are influenced by a reciprocal interaction between personal, behavioral, and environmental factors. SCT emphasizes the following key components: perceived self-efficacy (the belief in one’s ability to engage in physical activity successfully, even in the face of challenges), outcome expectations (the anticipated positive and negative consequences of engaging in physical activity), goal-setting (specific and achievable objectives for physical activity behavior), and sociocultural factors (environmental influences that support or hinder physical activity, such as access to facilities, social support, and time constraints) (Bandura, 2004).

**Figure 1:**
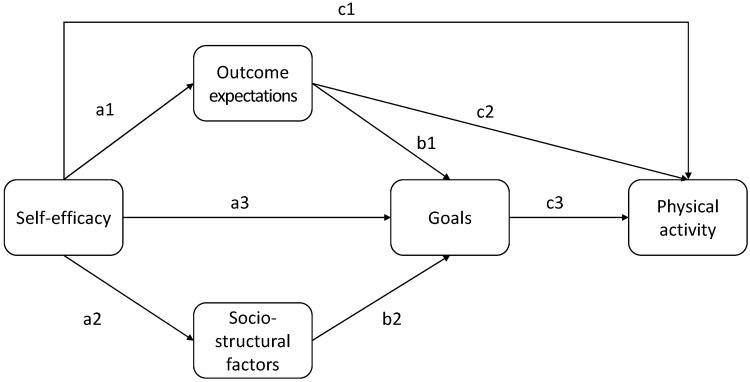
Schematic representation of the relationships between the constructs of social cognitive theory, as proposed by Bandura (2000).

Bandura (2000) suggests that self-efficacy is the most critical determinant of physical activity behavior. High self-efficacy leads to stronger outcome expectations, more concrete goals, and greater engagement in physical activity. Individuals with high self-efficacy are more likely to persist in physical activity despite obstacles and setbacks. The theoretical model is shown in Figure 1.

Previous studies confirmed the central role of self-efficacy and pointed to its direct and indirect effects on physical activity behavior (see, for example, Young et al., 2014 meta-analysis). The role of outcome expectations as a mediator in the SCT model has also been confirmed (Tulloch et al., 2020; Wójcicki et al., 2009). Furthermore, it can be inferred from previous empirical findings that goal setting has a direct effect on physical activity behavior (Zechner & Gill, 2016), and there is evidence that social environmental factors, which operate from the outside, also influence physical activity (Gothe, 2018; Hamilton et al., 2017; Sweeney et al., 2017).

However, evidence for the predictive and explanatory power of SCT concerning physical activity was shown predominantly for specific samples, for example, populations with diseases such as multiple sclerosis (e.g., Baird et al., 2021; Silveira & Motl, 2019), type 2 diabetes (e.g., Sebastian et al., 2021), severe mental illness (e.g., Zechner & Gill, 2016), cancer (e.g., Auster-Gussman et al., 2022; Stacey et al., 2015), and obesity (e.g., Young et al., 2016). Relatively little research addresses healthy adult populations (Young et al., 2014).

Secondly, SCT has rarely been fully tested and modeled, as intended by Bandura. Bandura himself criticized this early on, as stated by Luszczynska and Schwarzer (2015, p. 229): “Some authors seem to believe that SCT is equivalent to self-efficacy theory (SET), provoking repeated statements by Bandura (1997) that SCT is not a ‘one-factor theory’”. Similarly, in their systematic review of studies concerning SCT in the context of physical activity, Young et al. (2014) criticize “the majority of SCT research has focused solely upon self-efficacy or examined self-efficacy in combination with only one or two other variables” (p. 985). Accordingly, the recommendations for research on SCT are as follows: “To generate more valid data regarding the utility of SCT to explain PA, it is crucial that future studies include measures for all constructs in appropriately specified structural equation models and report the direct, indirect, and total effects of all variables.” (Young et al., 2016, p. N184).

Thirdly, there is a lack of evidence on the explanatory and predictive power of SCT concerning physical activity in Germany. To the best of our knowledge, there is exactly one study that has this focus. However, this study by Gellert et al. (2012) examined older adults aged 60–95 years and focused on affective and health-related outcome expectations. This, in turn, meets points 1 and 2 of the above-mentioned critique and confirms the need for a study on the explanatory power of SCT in the healthy general population in Germany that considers all the constructs and relationships between the elements envisaged by Bandura and verifies their tenability.

Therefore, the present study centers on three lines of inquiry:

We assume that the model fits the data and that it describes the theoretically stated relations between self-efficacy, outcome expectations, socio-structural factors, goal setting, and physical activity in its entity (RQ1).

Additionally, we examine the direct effect of self-efficacy on physical activity, as well as the direct effects of outcome expectations and goal setting on physical activity (RQ2).

Furthermore, we investigate the indirect effects of self-efficacy, outcome expectations, and socio-structural factors on physical activity (RQ3).

## 2. Materials and Methods

### 2.1. Sample and Procedure

Participants were recruited by posting notices on campus and in cultural centers in Saarland, Germany, in addition to placing online advertisements. The study was conducted on subjects between 18 and 64 years of age who had a command of the German language at the native speaker level and who did not have any health restrictions that would preclude participation in physical activity. Prior to analysis, data from subjects under the age of 18 (*n* = 2) or over the age of 64 (*n* = 3) were excluded. The survey was terminated for respondents indicating a lower language proficiency than the established requirement (*n* = 1) or reporting physical limitations (*n* = 0). Consequently, the data from these participants was not incorporated into the analyses.

**Figure 2:**
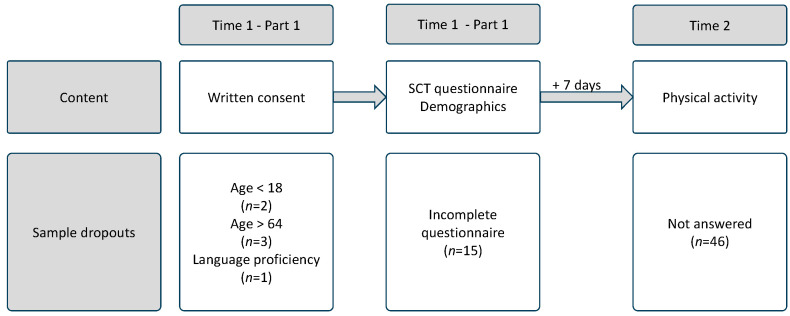
Course of the data collection.

The survey was fully conducted online with SosciSurvey (Leiner, 2024). Accordingly, a self-selected random sample was available for analysis in this study. Figure 2 depicts the course of the data collection. Data collection comprised two questionnaires. Prior to their participation in this study, all subjects provided written informed consent. In the initial questionnaire, subjects were instructed to provide answers regarding their demographic data, and their expressions on the SCT scale were assessed. The second point of measurement occurred seven days later to assess physical activity. The analyses were conducted on a dataset that exclusively comprised responses from subjects who had completed both questionnaires. Consequently, subjects who had only completed the first questionnaire (*n* = 46) or had discontinued the completion of the first (*n* = 15) or second (*n* = 0) questionnaire were excluded from the analyses.

The final sample consisted of 194 (109 male, 85 female) participants with a mean age of 26.03 years (SD = 10.33). The average amount of physical activity per week in the sample was 2478.30 MET minutes (SD = 2687.70). Although this is considerably more than 600 MET minutes of total physical activity per week, which is recommended by the WHO, it is not unusually high. For example, the national report for Germany shows that 85% of Germans report exercising for much more than 600 MET minutes (Der DKV-Report|DKV, 2023), and international studies also show that this level of activity is not unusual (Kyu et al., 2016).

### 2.2. Measures

Scales to measure the SCT components: A validated German scale to specifically assess the SCT components in the context of physical activity was applied (Egele & Stark, 2024). The scale consists of 18 items and has appropriate psychometric properties. We used an 11-point rating scale ranging from 0 (do not agree at all) to 10 (fully agree) for all items. Self-efficacy was measured by five items, its reliability was ω = 0.904. Outcome expectations were also measured by five items, and their reliability was ω = 0.776. The scale to measure goals comprised four items and its reliability was ω = 0.891. Finally, social cognitive factors were measured by four items, with a reliability of ω = 0.741.

Physical activity: Physical activity was assessed using the International Physical Activity Questionnaire—Short Form (Booth et al., 1996). The IPAQ-SF is a retrospective self-report questionnaire in which respondents rate their physical activity over the past seven days. The questions record the number of days with physical activity and the average time (measured in minutes) spent walking, performing moderate, and vigorous physical activity in an open-ended response format. All items for moderate and vigorous physical activity were presented with additional descriptions of example activities to foster a better understanding. We calculated the metabolic equivalent of task (MET) according to the formulas provided in the IPAQ manual. To achieve a total score of physical activity, the METs for walking, moderate, and vigorous physical activity were added. The resulting score was multiplied by the factor 1/1000 to avoid too large variances.

### 2.3. Statistical Analysis

All calculations were performed in R (R Core Team, 2020) using the packages lavaan (Rosseel, 2012) and psych (Revelle, 2023). To test our hypotheses, we set up the model shown in Figure 1 as a structural equation model (SEM) with latent variables. The SEM is composed of a measurement model part and a structural model part, the latter of which describes the theoretically stated relations between the individual constructs in SCT. Physical activity in terms of MET was included as a manifest variable. Because some of the latent variables’ indicators and MET had skewed distributions, we used the MLM estimator with the Satorra–Bentler-scaled χ^2^-test statistic and robust standard errors. Indirect and total effects were implemented through defined parameters.

To investigate the RQ1, we considered the model fit. Firstly, we investigated the measurement model of each latent variable separately with a one-factor confirmatory factor analysis (CFA). Afterwards, we also investigated a complete CFA model with all latent variables to investigate the model fit of the whole measurement model. The model fit was evaluated according to the criteria of Schermelleh-Engel et al. (2003). According to this classification, a model with a good fit should have a χ^2^-test statistic with a *p*-value greater than 0.05, and the RMSEA should be smaller than 0.05 with a *p*-value for the test of close fit greater than 0.10. For CFI, a value greater than 0.97, and for the SRMR, a value smaller than 0.05 reflects a good model fit.

Secondly, we estimated the SEM. The model with evaluation was performed with the before-mentioned criteria. The SEM’s fit measures are global measures that simultaneously assess the fit of the measurement and the structural part of the SEM and thus do not allow the separate assessment of the SEM’s structural part that consists of the theoretically stated relations (Lance et al., 2016). To obtain an evaluation of the structural part, Lance et al. (2016) propose to consider the C9 and C10 indices. The C9 index reflects a model comparison in which the assumed theoretical model is compared to a model that specifies no directional relationships among the latent variables, i.e., a model that only contains the latent variables’ variances. The C9 index is a goodness-of-fit index that ranges between 0 and 1, i.e., the closer its value to 1, the better the structural model’s fit. The C10 index refers to the absence of direct causal effects between specified variables, and stems from a model comparison between the assumed theoretical model and a model that contains the paths that were omitted in the theoretical model, which in our case is a model that contains a path between social–structural factors and physical activity. The C10 index is a badness-of-fit index that ranges between 0 and 1, i.e., the closer its value to 0 the better the structural model’s fit. To calculate the C9 and C10 indices, we used the formulas provided by Lance et al. (2016) that rely on the model’s χ^2^-test statistics. Additionally, to obtain some indications concerning the usual fit indices, we fitted a further CFA model that contained the latent variables’ covariances with MET. We used this model’s covariance matrix to estimate a structural model part only. Hereafter, this model is referred to as a partial structural model. For this estimation, we used the ML estimator to obtain the usual χ^2^-test statistic and the usual fit indices.

To investigate RQ2, we refer to the unstandardized path coefficients between the constructs of SCT and physical activity. For RQ3, we calculated the indirect effects (products of the path coefficients) and total effects (sum of direct and indirect effects).

For all effects, we also reported the standardized coefficients. All significance tests referring to RQ2 and RQ3 were conducted with a nominal significance level of α = 0.05. Data are available from the corresponding author upon reasonable request.

## 3. Results

Table 1 shows the correlational analyses of the latent variables with physical activity. A close examination reveals a robust correlation between self-efficacy and outcome expectations, both with respect to goals and physical activity behavior. Notably, goals and physical activity behavior exhibit a lack of significant correlation with each other, as well as with sociostructural factors.

### 3.1. Results Concerning Research Question 1

The results concerning RQ1 are presented in Table 2. The one-factorial measurement models and the complete CFA models indicate a good fit for each model. The SEM also indicates a good global model fit for the combined measurement and structural model parts. Concerning the separate assessment of the structural model part, the C9 index is 0.999, which hints at an exceptionally good fit for the structural model part. The C10 index, which is 0.0013, indicates the same conclusion. Thus, the structural part separated from the measurement model also indicates a good fit. To sum up, the investigation provides evidence for the relations as stated in the SCT.

### 3.2. Results Concerning Research Question 2

**Figure 3:**
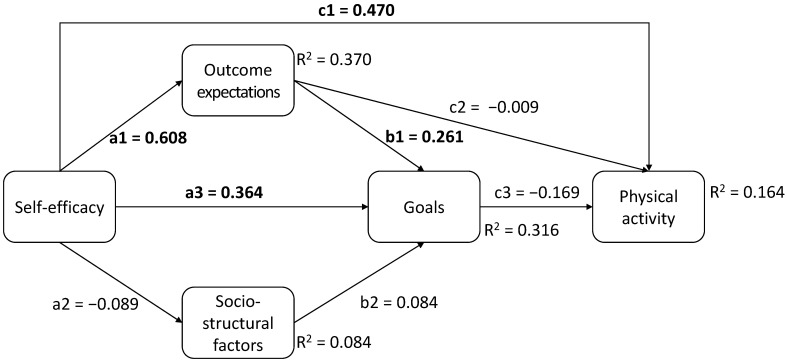
Path coefficients. Note: significant paths are marked in bold.

Table 3 (upper panel) presents the results concerning the direct effects; see also Figure 1 for the names of the path coefficients, and Figure 3 for a path model depicting the standardized coefficients and the explained variance. The results indicate that there are significant direct effects between self-efficacy and outcome expectations (path a1), self-efficacy and goals (path a3), outcome expectations and goals (path b1), as well as self-efficacy and physical activity (path c1).

From an interpretative perspective, the results indicate, in combination with the good model fit of the structural part, that relations described by SCT are valid. However, some insignificant direct paths indicate that not all theoretically described relations are important, for example, the direct effect of self-efficacy on socio-structural factors, of socio-structural factors on goals, and of outcome expectations on physical activity.

### 3.3. Results Concerning Research Question 3

Concerning the indirect and total effects, the results are shown in Table 3 (lower panel). Only one indirect effect is statistically significant, namely the path from self-efficacy via outcome expectations to goals (path a1*b1). This result is in line with the results concerning the direct effects. The results indicate that the theoretically assumed relations seem valid but unimportant.

## 4. Discussion

### 4.1. Interpretation of Findings

This study aimed to examine the predictive power of SCT concerning physical activity behavior in a non-clinical sample. Therefore, all of Bandura’s (2004) assumed relationships between the elements were taken into consideration, which has rarely been performed in the past. The present study makes a significant contribution to the field, as it employs an innovative analytical approach by considering the structural model independently from the measurement model. This enabled us to provide substantiated insights into the theoretical fit of Banduras assumptions concerning the relations of the constructs of SCT. The differentiation between the structural model and the measurement model is pivotal in facilitating the acquisition of substantiated insights into the validity of Bandura’s assumptions concerning the relations of the constructs of SCT. The prevailing standard of the SEM procedure is characterized by its inability to differentiate between these models (Lance et al., 2016). As a consequence, the procedure fails to enable the independent evaluation of theoretical assumptions vis à vis the measurement assumptions, thus impeding a comprehensive and systematic examination of the theoretical underpinnings. Results showed the very good fit of the structural model, indicating that the theoretically stated relations between the constructs in SCT seem corroborated. However, it seems that some of the theoretically assumed relations are less important than others despite being valid, which will be discussed in the following.

Taking a closer look at the results, the significant direct effect of self-efficacy on physical activity in the present study joins many similar findings in the literature. This finding also corresponds to the fact that many interventions that promote physical activity behavior focus on self-efficacy (Tang et al., 2019).

The indirect effect of self-efficacy on physical activity is rarely reported, and in the cases where it is reported, evidence for significance is often absent, as in the present study. Perhaps the complexity of the indirect effect plays a role here, as it includes several paths. Given the analysis of the individual pathways that follow later, which together result in the indirect effect of self-efficacy on physical activity, the question arises whether some of these pathways are more relevant than others. Subsequently, it could be considered if the assumed indirect effect could be slimmed down, i.e., whether it is possible to distance oneself from the assumption of an all-encompassing indirect effect. A second consideration, however, is the relevance of the indirect effect of self-efficacy on physical activity against the background of the direct effect of self-efficacy on physical activity being so well documented. The question arises to what extent a potential indirect effect is of importance if a direct effect on physical activity behavior can be achieved in interventions by promoting self-efficacy.

As suggested by previous findings, outcome expectations would have a significant direct effect on physical activity behavior in our study. However, in the Young et al. (2014) meta-analysis, a positive and significant effect of outcome expectations on physical activity behavior was reported in about 30% of studies that examined the effect of outcome expectancies on physical activity. Our findings may contribute to the body of studies that investigated the effect but have been unable to prove it. It is similarly conceivable that this finding can be attributed to the measurement of outcome expectations, given that only positive outcome expectations were queried on the scale employed. Perhaps the impact of negative action outcome expectations on exercise behavior is more pronounced than that of positive experiences, explaining the non-significant relation. Alternatively, it would also be plausible that outcome expectations are of greater importance for individuals with lower levels of physical activity than for those who are more active. As the present sample was already active, it is possible that outcome expectations were not the deciding factor for physical activity. Therefore, it seems relevant to examine which factors influence the occurrence and the direction of the effect.

A significant indirect effect of outcome expectations on physical activity is rarely reported (c.f. Young et al., 2014). No evidence for this indirect effect was found in the present study either. However, the fact that goals had no significant effect on physical activity behavior in the present study (see the next section)—in contrast to previous findings in the literature—may also have had an influence. Studies that report an indirect effect of outcome expectations on physical activity like Gellert et al. (2012) demonstrate an indirect pathway mediated by “intention”. Goals may be conceptualized more broadly than intentions, which might explain why the effect was not significant in our study.

Goals are usually consistently associated with physical activity behavior in the literature. In this study, the effect was not significant, hinting at a possible intention–behavior gap. The absence of a discernible correlation is further substantiated by the bivariate correlation analysis of goals and exercise behavior. The absence of a substantial correlation could be ascribed, at least in part, to the item formulation. Nevertheless, the observation that goals are closely associated with self-efficacy and outcome expectations provides a compelling counterargument to this assertion. Consequently, it can be deduced that a discordance exists between goals and physical activity behavior, as recorded in this study. Future studies should re-examine this relation, especially as more recent research results also show that it might be beneficial to assess goals more specifically tailored, i.e., adapted, to the type, quantity, and intensity of the respective physical activity (Conner & Norman, 2022).

Bandura does not include the direct effect of socio-structural factors on behavior. This restriction seems appropriate, as the structural model fit with this restriction was good. The fact that also no indirect effect of socio-structural factors on physical activity behavior was found is consistent with previous findings reporting that socio-structural factors do not seem to have a significant indirect effect on physical activity. Nevertheless, the question arises to what extent socio-structural factors play a role in physical activity behavior if neither a direct nor an indirect influence on physical activity behavior can be confirmed. Potentially, it would be beneficial to conduct moderator analyses to ascertain whether the effect is present under specific circumstances.

At this juncture, a discussion of the correlation matrix of the social–cognitive factors with each other, as well as with the criterion, is warranted, with a special focus on the socio-structural factors. While the majority of the bivariate correlations are consistent, it is evident that the socio-structural factors do not exhibit a significant correlation with any other construct. This may be attributable to the heterogeneity of the construct; alternatively, it is plausible that the sample utilized led to a restriction of variance in this domain. Notwithstanding, given the absence of a statistically significant correlation between the socio-structural factors and physical activity behavior, it is not unexpected that the model with a corresponding path restriction exhibits a satisfactory fit.

Overall, our findings are broadly consistent with previous findings, supporting the utility of SCT to explain physical activity. Yet, some differences between the present findings and common previous findings need to be discussed. 

Despite a good model fit, the variance explained in physical activity behavior was smaller than in similar studies (e.g., meta-analysis by Young et al., 2014: R2 = 0.30). Multiple aspects can be mentioned. Firstly, meta-analyses show that the average age of the subjects is a moderator of the variance explained in the criterion. While more variance can be explained in older subjects (Young et al., 2014), the relatively young average age in the present sample could be one explanation for the smaller variance explained. Secondly, the homogeneity of our sample may have limited the extent to which the observed variance could be explained. Finally, the criterion of physical activity behavior was recorded employing IPAQ. The IPAQ is a common tool for recording physical activity, but is not free of criticism—for example, it is susceptible to bias based on self-reporting (Egele et al., 2021; Straßburg et al., 2019). The IPAQ captures a total physical activity score, which includes vigorous and moderate physical activity as well as walking. While several studies use a similar criterion, “a measure of total PA” (Young et al., 2014, p. 987), some have also captured physical activity more precisely and more objectively, such as via a number of steps. Therefore, the criterion capture per se may have an impact on the variance explained.

It also seems important to consider that behavior often proves less predictable than the goals or the intention to exhibit certain behaviors. This phenomenon, referred to as the “intention–behavior gap”, is a well-documented occurrence, especially in the context of physical activity (Feil et al., 2023), and was also shown in the present study. While the variance explained in physical activity was rather small, a far larger share of the variance of goals could be explained. Meta-analyses show that while intentions or goals seem necessary for behavior, it seems like the probability of translating this intention into behavior is random at best (Feil et al., 2023; Rhodes & de Bruijn, 2013). Recent research shows that assessing not only goals but also the strength or stability of these goals could lead to a more accurate explanation of behavior (Conner & Norman, 2022). This was not explicitly included in Bandura’s goal construct but might be worth adding in the future to reduce the intention–behavior gap.

If we consider the findings and their classification in the previous literature and research, we conclude that SCT seems to be suitable to explain physical activity behavior in a healthy sample. However, more research is needed to delve deeper into the indirect pathways and underlying mechanisms that influence physical activity behavior within the SCT framework.

In addition, potential moderators (e.g., age, sex, physical activity level, and other personal characteristics) should be investigated regarding their effect on the adequacy of the theoretical assumptions.

### 4.2. Limitations

It is important to note that the representativeness of the sample may be limited by the distorted gender ratio. Consequently, the results should be replicated in further studies to secure external validity. Further studies could illuminate the question of whether some paths are truly non-significant in a non-clinical sample, as found in the present study, or whether sample characteristics lead to non-significant paths. Furthermore, to generalize the results, it would be beneficial to replicate the study with a criterion that is more objectively recorded.

Another possible issue is the sample size. Typical recommendations for the MLM estimator are sample sizes of 250 or greater (e.g., Hu & Bentler, 1999). Our sample only amounts to 194 participants, so it undercuts the recommendation by about 22%. A possible problem with a smaller sample size than recommended is overcorrecting the standard errors. However, this issue does not likely affect our results, as we did not find as many significant paths as theoretically expected. In this respect, we want to point out that such sample size boundaries are only recommendations that should not be considered definitive. Nonetheless, the sample size may have constrained the capacity to discern potentially substantial pathways. This limitation could serve as a rationale for the observed insignificance of certain pathways.

Additionally, based on our findings, we cannot determine whether the good fit of the theoretical assumptions of SCT actually corresponds to the interaction of the constructs. Recently, for example, the directionality of the paths has been increasingly questioned (e.g., Beauchamp et al., 2019) and this matter cannot be clarified based on the data of this study.

### 4.3. Theoretical and Practical Implications

The present study confirms the tenability of the theoretical assumptions of social-cognitive theory as set out by Bandura. This conclusion seems particularly solid due to the distinction made between the structural model and the measurement model, which enabled the provision of substantiated insights into the theoretical fit of Bandura’s assumptions concerning the relations of the constructs of SCT. The standard SEM procedure fails to differentiate between the structural and measurement models, thereby precluding the independent evaluation of theoretical assumptions against the measurement assumptions. In contrast, the approach adopted in this study to address the specified research question is commendable. The verification of the theoretical assumptions is of paramount importance, given the persistent utilization of social–cognitive theory as a foundational framework for behavioral explanation and modification studies (Luszczynska & Schwarzer, 2015). Consequently, it was imperative to ascertain the empirical validity of the theoretically postulated relations.

Despite the ostensible validity of the theoretical underpinnings of SCT, it has been demonstrated that certain pathways hold greater significance than others. At the forefront of this discourse lies the role of self-efficacy, which exerts a strong direct influence on behavior. The significant direct impact of self-efficacy on behavior, as compared to the seemingly lesser relevance of alternate pathways, prompts the examination of whether the implementation of SCT is warranted. Alternatively, the inquiry extends to whether the adoption of self-efficacy theory (Bandura, 1977) as a fundamental framework might yield equivalent outcomes.

The validation of the theoretical assumptions underlying SCT has significant ramifications for the field of practice. For instance, further studies grounded in SCT can be pursued to elucidate and modify behaviors. It can be contended that the present findings provide a robust basis for the initiation of additional studies, as a prevailing criticism of the theory—namely, the absence of a holistic model (Beyera et al., 2021)—has been addressed. In light of our findings, we would advocate for the prominent role of self-efficacy as a potential starting point for behavior change interventions and recommend targeting it due to its strong effect. Behavior change research offers numerous approaches for fostering self-efficacy concerning physical activity (e.g., Stojanovic et al., 2021; Warner & Schwarzer, 2020). The findings of this study can also be significant in everyday life, both for health practitioners and for individuals. For instance, the role of mastery experiences, which have been demonstrated to influence self-efficacy (Wiedenman et al., 2024), can be leveraged to enhance self-efficacy, which in turn can impact physical activity behavior. Analogous considerations can be drawn when examining outcome expectations; it may be feasible to deliberately focus on positive outcome expectations in order to manifest a desired behavior. Consequently, the present study provides important theoretical implications while also highlighting potential practical consequences.

## 5. Conclusions

The current study supports previous studies finding that SCT is useful in explaining physical activity behavior. Previous findings were extended by a holistic examination of the theory and a specific focus on the fit of the structural model assumed by Bandura. Nonetheless, previous studies that only investigated selected elements of the theory were also corroborated in that this study demonstrated the greater importance of certain model elements, including, in particular, self-efficacy and expected outcomes.

## Figures and Tables

**Table 1:** Correlations of the latent variables with physical activity.

	Physical Activity	Self- Efficacy	Goals	Outcome Expectations	Sociostructural Factors
Physical activity	-				
Self-efficacy	0.378 ***	-			
Goals	0.069	0.516 ***	-		
Outcome expectations	0.196 ***	0.608 ***	0.479 ***	-	
Sociostructural factors	−0.021	−0.091	0.041	−0.040	-

Note. *** *p* < 0.001.

**Table 2:** Fit measure of the measurement and structural equation models.

	χ^2^	*df*	*p*	CFI	SRMR	RMSEA	*p*_close_
Self-efficacy	4.262	5	0.512	1.000	0.018	0.000	0.822
Goals	0.487	2	0.784	1.000	0.007	0.000	0.918
Outcome expectations	3.801	5	0.578	1.000	0.027	0.000	0.846
Socio-structural factors	1.451	2	0.484	1.000	0.023	0.000	0.689
Complete CFA	148.477	129	0.116	0.986	0.079	0.028	0.986
SEM	159.284	145	0.197	0.990	0.077	0.023	0.997
Null model *	294.195	-	-	-	-	-	-
All paths SEM *	158.795	-	-	-	-	-	-
Partial structural model	0.264	2	0.876	1.000	0.008	0.000	0.922

Note. * The null model and the all-paths SEM are used for the calculation of the C9 and C10 indices. For these calculations, the χ^2^-test statistic and the models as such are not judged concerning their model fit. Therefore, the table only reports the χ^2^-test statistics.

**Table 3:** Direct, indirect, and total effects for the standardized regression coefficients.

Paths	Estimate	*SE*	*z*	*p*
Total effect				
Total effect	**0.377**	0.057	6.661	<0.001
Direct effects (RQ2)				
a1	**0.608**	0.060	10.175	<0.001
a2	−0.089	0.083	−1.073	0.283
a3	**0.364**	0.095	3.824	<0.001
b1	**0.261**	0.107	2.441	0.015
b2	0.084	0.071	1.180	0.238
c1	**0.470**	0.107	4.391	<0.001
c2	−0.009	0.093	−0.095	0.924
c3	−0.169	0.095	−1.774	0.076
Indirect effects (RQ3)				
a1*c2	−0.005	0.057	−0.095	0.924
a1*b1	**0.159**	0.067	2.383	0.017
a1*b1*c3	−0.027	0.020	−1.324	0.186
a3*c3	−0.061	0.035	−1.747	0.081
a2*b2	−0.007	0.010	−0.717	0.474
a2*b2*c3	0.001	0.002	0.639	0.523
b1*c3	−0.044	0.033	−1.330	0.184
b2*c3	−0.014	0.015	−0.936	0.349
a1*c2+ a1*b1+c3+a2*b2*c3+a3*c3	−0.092	0.075	−1.237	0.216

Note. See Figure 1 for the designation of the path coefficients. All significance tests referring to RQ2 and RQ3 were conducted with a nominal significance level of α = 0.05. Significant paths are marked in bold.

## Data Availability

Data are available from the authors upon request.

## References

[B1-ejihpe-15-00020] Auster-Gussman L. A., Gavin K. L., Siddique J., Welch W. A., Solk P., Whitaker M., Cullather E., Fanning J., Maria C. S., Gradishar W., Khan S., Kulkarni S., Phillips S. M. (2022). Social cognitive variables and physical activity during chemotherapy for breast cancer: An intensive longitudinal examination. Psycho-Oncology.

[B2-ejihpe-15-00020] Baird J. F., Silveira S. L., Motl R. W. (2021). Social cognitive theory and physical activity in older adults with multiple sclerosis. International Journal of MS Care.

[B3-ejihpe-15-00020] Bandura A. (1977). Self-efficacy: Toward a unifying theory of behavioral change. Psychological Review.

[B4-ejihpe-15-00020] Bandura A. (1986). Social foundations of thought and action: A social cognitive theory.

[B5-ejihpe-15-00020] Bandura A. (1997). Self-efficacy: The exercise of control.

[B6-ejihpe-15-00020] Bandura A., Abraham C., Norman P., Conner M. (2000). Health promotion from the perspective of social cognitive theory. Understanding and changing health behaviour: From health beliefs to self-regulation.

[B7-ejihpe-15-00020] Bandura A. (2004). Health promotion by social cognitive means. Health Education & Behavior.

[B8-ejihpe-15-00020] Beauchamp M. R., Crawford K. L., Jackson B. (2019). Social cognitive theory and physical activity: Mechanisms of behavior change, critique, and legacy. Psychology of Sport and Exercise.

[B9-ejihpe-15-00020] Beyera G. K., O’Brien J., Campbell S. (2021). Choosing a health behaviour theory or model for related research projects: A narrative review. Journal of Research in Nursing.

[B10-ejihpe-15-00020] Booth M. L., Owen N., Bauman A. E., Gore C. J. (1996). Retest reliability of recall measures of leisure-time physical activity in Australian adults. International Journal of Epidemiology.

[B11-ejihpe-15-00020] Conner M., Norman P. (2022). Understanding the intention-behavior gap: The role of intention strength. Frontiers in Psychology.

[B12-ejihpe-15-00020] Der DKV-Report|DKV (2023). https://www.dkv.com/der-dkv-report.html.

[B13-ejihpe-15-00020] Egele V. S., Stark R. (2024). Operationalization of the social cognitive theory to explain and predict physical activity in Germany: A scale development. Frontiers in Sports and Active Living.

[B14-ejihpe-15-00020] Egele V. S., Kiefer L. H., Stark R. (2021). Faking self-reports of health behavior: A comparison between a within-and a between-subjects design. Health Psychology and Behavioral Medicine.

[B15-ejihpe-15-00020] Feil K., Fritsch J., Rhodes R. E. (2023). The intention-behaviour gap in physical activity: A systematic review and meta-analysis of the action control framework. British Journal of Sports Medicine.

[B16-ejihpe-15-00020] Gellert P., Ziegelmann J. P., Schwarzer R. (2012). Affective and health-related outcome expectancies for physical activity in older adults. Psychology & Health.

[B17-ejihpe-15-00020] Gothe N. P. (2018). Correlates of physical activity in urban African American adults and older adults: Testing the social cognitive theory. Annals of Behavioral Medicine.

[B18-ejihpe-15-00020] Hamilton K., Warner L. M., Schwarzer R. (2017). The role of self-efficacy and friend support on adolescent vigorous physical activity. Health Education & Behavior.

[B19-ejihpe-15-00020] Heiss V. J., Petosa R. L. (2016). Social cognitive theory correlates of moderate-intensity exercise among adults with type 2 diabetes. Psychology, Health & Medicine.

[B20-ejihpe-15-00020] Hu L. T., Bentler P. M. (1999). Cutoff criteria for fit indexes in covariance structure analysis: Conventional criteria versus new alternatives. Structural Equation Modeling: A Multidisciplinary Journal.

[B21-ejihpe-15-00020] Kyu H. H., Bachman V. F., Alexander L. T., Mumford J. E., Afshin A., Estep K., Veerman J. L., Delwiche K., Iannarone M. L., Moyer M. L., Cercy K., Vos T., Murray C. J. L., Forouzanfar M. H. (2016). Physical activity and risk of breast cancer, colon cancer, diabetes, ischemic heart disease, and ischemic stroke events: Systematic review and dose-response meta-analysis for the Global Burden of Disease Study 2013. BMJ.

[B22-ejihpe-15-00020] Lance C. E., Beck S. S., Fan Y., Carter N. T. (2016). A taxonomy of path-related goodness-of-fit indices and recommended criterion values. Psychological Methods.

[B23-ejihpe-15-00020] Leiner D. J. (2024). SoSci Survey *(Version 3.1.06) [Computer software]*.

[B24-ejihpe-15-00020] Luszczynska A., Schwarzer R. K. (2015). Social cognitive theory.

[B25-ejihpe-15-00020] Miko H.-C., Zillmann N., Ring-Dimitriou S., Dorner T. E., Titze S., Bauer R. (2020). Auswirkungen von Bewegung auf die Gesundheit. Das Gesundheitswesen.

[B26-ejihpe-15-00020] R Core Team (2020). European Environment Agency. *(n.d.). [Methodology Reference]*.

[B27-ejihpe-15-00020] Revelle W. (2023). psych: Procedures for psychological, psychometric, and personality research *(2.3.3) [Computer software]*.

[B28-ejihpe-15-00020] Rhodes R. E., de Bruijn G.-J. (2013). How big is the physical activity intention–behaviour gap? A meta-analysis using the action control framework. British Journal of Health Psychology.

[B29-ejihpe-15-00020] Rosseel Y. (2012). lavaan: An R package for structural equation modeling. Journal of Statistical Software.

[B30-ejihpe-15-00020] Schermelleh-Engel K., Moosbrugger H., Müller H. (2003). Evaluating the fit of structural equation models: Tests of significance and descriptive goodness-of-fit measures. Methods of Psychological Research.

[B31-ejihpe-15-00020] Sebastian A. T., Rajkumar E., Tejaswini P., Lakshmi R., Romate J. (2021). Applying social cognitive theory to predict physical activity and dietary behavior among patients with type-2 diabetes. Health Psychology Research.

[B32-ejihpe-15-00020] Silveira S. L., Motl R. W. (2019). Do Social Cognitive Theory constructs explain response heterogeneity with a physical activity behavioral intervention in multiple sclerosis?. Contemporary Clinical Trials Communications.

[B33-ejihpe-15-00020] Stacey F. G., James E. L., Chapman K., Courneya K. S., Lubans D. R. (2015). A systematic review and meta-analysis of social cognitive theory-based physical activity and/or nutrition behavior change interventions for cancer survivors. Journal of Cancer Survivorship: Research and Practice.

[B34-ejihpe-15-00020] Stojanovic M., Fries S., Grund A. (2021). Self-Efficacy in habit building: How general and habit-specific self-efficacy influence behavioral automatization and motivational interference. Frontiers in Psychology.

[B35-ejihpe-15-00020] Strain T., Flaxman S., Guthold R., Semenova E., Cowan M., Riley L. M., Bull F. C., Stevens G. A. (2024). National, regional, and global trends in insufficient physical activity among adults from 2000 to 2022: A pooled analysis of 507 population-based surveys with 5· 7 million participants. The Lancet Global Health.

[B36-ejihpe-15-00020] Straßburg A., Eisinger-Watzl M., Krems C., Roth A., Hoffmann I. (2019). Comparison of food consumption and nutrient intake assessed with three dietary assessment methods: Results of the German National Nutrition Survey II. European Journal of Nutrition.

[B37-ejihpe-15-00020] Sweeney A. M., Wilson D. K., Lee, Van Horn M. (2017). Longitudinal relationships between self-concept for physical activity and neighborhood social life as predictors of physical activity among older African American adults. International Journal of Behavioral Nutrition and Physical Activity.

[B38-ejihpe-15-00020] Tang M. Y., Smith D. M., Mc Sharry J., Hann M., French D. P. (2019). Behavior change techniques associated with changes in postintervention and maintained changes in self-efficacy for physical activity: A systematic review with meta-analysis. Annals of Behavioral Medicine.

[B39-ejihpe-15-00020] Taylor L. M., Raine K. D., Plotnikoff R. C., Vallance J. K., Sharma A. M., Spence J. C. (2016). Understanding physical activity in individuals with prediabetes: An application of social cognitive theory. Psychology, Health & Medicine.

[B40-ejihpe-15-00020] Tulloch H., Heenan A., Sweet S., Goldfield G. S., Kenny G. P., Alberga A. S., Sigal R. J. (2020). Depressive symptoms, perceived stress, self-efficacy, and outcome expectations: Predict fitness among adolescents with obesity. Journal of Health Psychology.

[B41-ejihpe-15-00020] Warner L. M., Schwarzer R., Sweeny K., Robbins M. L., Cohen L. M. (2020). Self-efficacy and health. The wiley encyclopedia of health psychology: Volume II, the social bases of health behavior.

[B42-ejihpe-15-00020] Wiedenman E. M., Kruse-Diehr A. J., Bice M. R., McDaniel J., Wallace J. P., Partridge J. A. (2024). The role of sport participation on exercise self-efficacy, psychological need satisfaction, and resilience among college freshmen. Journal of American College Health.

[B44-ejihpe-15-00020] World Health Organization (2018). Global action plan on physical activity 2018–2030: More active people for a healthier world.

[B43-ejihpe-15-00020] Wójcicki T. R., White S. M., McAuley E. (2009). Assessing outcome expectations in older adults: The multidimensional outcome expectations for exercise scale. The Journals of Gerontology: Series B.

[B45-ejihpe-15-00020] Young M. D., Plotnikoff R. C., Collins C. E., Callister R., Morgan P. J. (2014). Social cognitive theory and physical activity: A systematic review and meta-analysis. Obesity Reviews.

[B46-ejihpe-15-00020] Young M. D., Plotnikoff R. C., Collins C. E., Callister R., Morgan P. J. (2016). A test of social cognitive theory to explain men’s physical activity during a gender-tailored weight loss program. American Journal of Men’s Health.

[B47-ejihpe-15-00020] Zechner M. R., Gill K. J. (2016). Predictors of physical activity in persons with mental illness: Testing a social cognitive model. Psychiatric Rehabilitation Journal.

